# RETRACTION: Parameter‐Based Transfer Learning for Severity Classification of Atopic Dermatitis Using Hyperspectral Imaging

**DOI:** 10.1111/srt.70281

**Published:** 2025-10-21

**RETRACTION**: E. B. Kim, Y. S. Baek, and O. Lee, “Parameter‐Based Transfer Learning for Severity Classification of Atopic Dermatitis Using Hyperspectral Imaging,” *Skin Research and Technology* 30, no. 4(2024):e13704, https://doi.org/10.1111/srt.13704.

The above article, published online on 16 April 2024 in Wiley Online Library (wileyonlinelibrary.com), has been retracted by agreement between *Skin Research and Technology*; and John Wiley & Sons Ltd. The article was accepted for publication following an insufficient peer review process that does not meet the standards of the journal's peer review policy. Furthermore, methodological inconsistencies were identified related to the use of images from the DermNet dataset (https://dermnetnz.org/) that compromise the reliability of the results obtained. Accordingly, the article has been retracted. The authors have been informed of the retraction decision but were unavailable for final confirmation.

